# Phenotypic Variation of Oak Species (*Quercus* spp.) Reveals Adaptive Strategies Across Natural and Semi‐Artificial Oak Stands

**DOI:** 10.1002/ece3.71217

**Published:** 2025-06-16

**Authors:** Xuan Li, Xuxu Bao, Yongfu Li, Yousry A. El‐Kassaby, Yanming Fang

**Affiliations:** ^1^ Co‐Innovation Center for Sustainable Forestry in Southern China, Key Laboratory of State Forestry and Grassland Administration on Subtropical Forest Biodiversity Conservation, College of Life Science Nanjing Forestry University Nanjing China; ^2^ Department of Forest and Conservation Sciences, Faculty of Forestry The University of British Columbia Vancouver British Columbia Canada; ^3^ Jiangsu Key Laboratory for the Research and Utilization of Plant Resources, Institute of Botany Jiangsu Province and Chinese Academy of Sciences (Nanjing Botanical Garden Mem. Sun Yat‐Sen) Nanjing Jiangsu China

**Keywords:** fluctuating asymmetry, phenotypic plasticity, phenotypic traits variation, *Quercus*

## Abstract

This study investigated leaf phenotypic variation in oak species to better understand how different groups of oaks adapt to diverse environmental conditions. We examined the leaf phenotypic traits of six oak populations in two mixed forests with differing species compositions: Zijin Mountain in Jiangsu Province, composed of *Quercus acutissima* , *Q. variabilis* , and *Q. fabri*; and Youhua Village in Anhui Province, consisting of *Q. acutissima* , *Q. chenii* , and *Q. fabri*. The results indicated that species in the *Cerris* group ( *Q. acutissima* , *Q. chenii*, and *Q. variabilis* ) exhibited stable leaf morphology and higher fluctuating asymmetry (FA), suggesting adaptation to stable environments. In contrast, *Q. fabri* from the *Quercus* group showed higher phenotypic plasticity and lower FA, indicating a strategy for adapting to dynamic environments. The study also explored the relationship between FA and phenotypic plasticity, revealing that while both traits are influenced by environmental stress, phenotypic plasticity allowed for more flexible responses to environmental change. Additionally, our research highlighted the role of hybridization and genetic coadaptation in influencing developmental stability, with higher hybridization rates in *Q. fabri* leading to greater morphological variability. These findings underscore the importance of environmental factors, genetic variation, and hybridization in shaping the adaptive strategies and phenotypic traits of oak species, providing valuable insights into the complexities of adaptation and species identification.

## Graphical abstract

Our findings highlight the significant impact of forest management on morphological traits, particularly the differences observed between the semi‐artificial forest at Zijin Mountain and the natural forest at Youhua Village. The study underscores the influence of both biotic and abiotic factors on phenotypic traits, with notable implications for forest management and conservation strategies. The observed relationship between FA and hybridization potential, especially in the context of the more variable natural forest environment, offers new insights into the adaptive strategies of oak species.

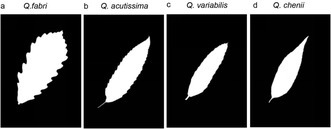

## 1 Introduction

Oak species (*Quercus* spp.) are a crucial component of temperate forests in the Northern Hemisphere, playing a key role in ecosystem stability and providing essential habitats for wildlife (Martinetto et al. 2020; Denk et al. 2017; Manos and Hipp 2021). In China, oaks (*Quercus* spp.) occupy a variety of ecological niches and serve as keystone species in numerous phytosociological classifications (Wu et al. 2019; Li et al. 2024a). Deciduous species like *Q. variabilis* , *Q. acutissima* , *Q. chenii* , and *Q. fabri* contribute significantly to soil conservation and ecological balance in various regions, ranging from temperate forests to subtropical zones (Li et al. 2019; Li et al. 2022; Niu et al. 2020; Chen et al. 2021). Understanding the adaptive strategies and phenotypic variation of these species is essential for exploring their roles in ecosystem functioning and their response to environmental challenges.

Leaves, as the primary organs for photosynthesis, respiration, and transpiration, directly impact the physiological functions and ecological adaptability of plants (Lopez and Barclay 2017). Leaf phenotypic variation results from the combined effects of genetic and environmental factors (Solé‐Medina et al. 2022; Long et al. 2011; Xu et al. 2023). Genetic factors determine the plant's genetic makeup and expression patterns, while environmental factors significantly influence leaf morphology by affecting the plant's growth conditions and physiological processes (Gong et al. 2023). For instance, in oaks, different species such as *Q. fabri* and *Q. variabilis* exhibit distinct leaf morphologies due to their genetic backgrounds (Li et al. 2022). Moreover, the phenomenon of hybridization in oaks further increases the diversity of leaf phenotypes. For example, hybrid offspring of *Q. fabri* and *Q. serrata* var. *Brevipetiolata* may exhibit a combination of the leaf characteristics of both parent species (Li et al. 2024b). This new variation resulting from hybridization provides plants with a broader adaptive potential in changing environments (Li et al. 2024b). Studying species' leaf phenotypes can enhance our understanding of their adaptation mechanisms under different environmental conditions and provide scientific bases for forest management and ecological conservation.

Traditional studies on leaf phenotypes have relied heavily on basic morphological measurements such as leaf length, width, and area (Zhao et al. 2022; Viscosi et al. 2009; Marcus 1990). While these methods have provided valuable insights, they often fall short in capturing the full complexity of leaf traits and their responses to environmental variations (Migicovsky et al. 2018). Moreover, these conventional methods are sometimes limited by subjective biases and measurement inconsistencies (Vincenzo 2015). In this study, we introduce an innovative approach to measuring leaf phenotypic traits using advanced geometric morphometric methods (GMMs). This technique allows for a more detailed and objective analysis of leaf morphology by quantifying both size and shape components, including the symmetric and asymmetric aspects of leaf structure (Akli et al. 2022; Liu et al. 2019; López‐De‐Heredia et al. 2018). GMMs provide a higher resolution of morphological variations, enabling the detection of subtle differences and patterns that those traditional methods might overlook. Furthermore, we employ fluctuating asymmetry (FA) analysis as an additional metric to assess leaves developmental stability (Albarrán‐Lara et al. 2010; Cuevas‐Reyes et al. 2018). FA measures the small, random deviations from perfect symmetry in bilaterally symmetrical traits, serving as an indicator of environmental and genetic stress (Albarrán‐Lara et al. 2010).

Phenotypic variation in species is driven by both environmental and genetic factors. The evolution of developmental instability (DI) is closely linked to genetic variation. While excessive DI may reduce adaptability, higher DI can become advantageous for coping with stress in fluctuating environments (Sangster et al. 2004). FA, often considered the primary manifestation of DI, represents random deviations from perfect symmetry in bilateral traits during development (Dongen 2006). Both FA and DI are responses to environmental or genetic disturbances and are central to understanding developmental processes (Graham 2021; Castillo and González‐Rivas 2023; Zakharov and Trofimov 2022). FA is typically assessed by measuring small deviations between bilateral traits, which reflect disturbances during development. External factors, such as environmental stress, can influence FA by affecting both developmental noise and DI, thus altering its level (Zakharov et al. 2020; Simbula et al. 2021; Zhelev et al. 2022). However, whether FA can accurately reflect individual fitness, particularly in terms of reproduction, survival, behavior, and physiology, remains an unresolved issue.

In contrast, phenotypic plasticity refers to an organism's ability to modify its phenotype in response to varying environmental conditions (Sjulgård et al. 2021). Unlike FA, which is an indicator of developmental instability, phenotypic plasticity reflects adaptive, reversible changes that enhance an organism's fitness under stress (Schneider 2022). While FA often signals developmental maladaptation or stress, phenotypic plasticity enables organisms to adjust morphologically or behaviorally to new or fluctuating environments, thereby improving their chances of survival and reproductive success (Xue and Leibler 2018). These plastic responses, ranging from subtle genetic changes to more noticeable modifications in morphology or physiology, are vital for coping with environmental challenges (Storz and Scott 2021). Although both FA and phenotypic plasticity are influenced by environmental stress, phenotypic plasticity is typically viewed as a more adaptive response, providing the flexibility needed to thrive in changing conditions. Together, these concepts provide complementary insights into how organisms adapt to environmental pressures.

Our research group has previously conducted a study on phenotypic variation in the mixed oak forests at Yushan Forest in Changshu, revealing that hybridization in oak trees leads to significant phenotypic variation, with hybrid individuals exhibiting adaptive phenotypic changes (Li, Zhang, et al. 2022). To further explore whether there are differences in phenotypic variation among mixed oak forests with different stand compositions, we plan to conduct a comparative study of phenotypic variation in two different sample plots. By comparing the phenotypic characteristics of individuals in different mixed oak forest plots, we aim to reveal the impact of stand composition on phenotypic variation and its adaptive mechanisms. This study aims to address three scientific questions: (1) What is the relationship between the trends in fluctuating asymmetry (FA) and phenotypic plasticity in species under different environmental conditions? (2) Are the adaptive strategies of the four oak species consistent? and (3) Does phenotypic variation affect species classification? The primary objective of this research is to systematically measure and analyze the leaf phenotypes of *Q. acutissima* , *Q. variabilis* , *Q. fabri*, and *Q. chenii* in two mixed forests in Jiangsu Province, revealing differences in leaf morphology and functional traits, as well as their ecological adaptation mechanisms.

## 2 Materials and Methods

### 2.1 Study Area and Sampling

This study was conducted in two mixed forests in Jiangsu and Anhui Province, China. The first site, located at Zijin Mountain (32.06° N, 118.87° E), features a semi‐artificial forest composed of *Q. acutissima* (*Cerris* group), *Q. variabilis* (*Cerris* group), and *Q. fabri* (*Quercus* group). The region experiences a northern subtropical monsoon climate with distinct seasons. Annual precipitation ranges from 1000 to 1050 mm, with an average annual temperature of 15.4°C and an average annual sunshine duration of 2213 h. The second site, situated at Youhua Village (30.68° N, 118.02° E), contains a natural forest with *Q. acutissima* , *Q. chenii* (*Cerris* group), and *Q. fabri*. The region belongs to the northern subtropical humid monsoon climate zone, dominated by the East Asian monsoon. It has an average annual temperature of 16.1°C and an average annual precipitation of 1558.6 mm. In total, 474 individual trees were sampled from the two study areas. At Zijin Mountain, we collected leaves from 37 *Q. acutissima* , 191 *Q. variabilis* , and 73 *Q. fabri* individuals, while at Youhua Village, we sampled 50 *Q. acutissima* , 82 *Q. chenii* , and 41 *Q. fabri* individuals (Table 1). For each tree, more than 10 healthy and mature leaves were gathered from different branches.

**TABLE 1:** Information of the two oaks mixed forests in Jiangsu province.

Populations/species	Code	Section	Site	Latitude (N)/longitude (E)	Sample size
*Q. acutissima*	ZJ_A	*Cerris*	Zijin mountain	118.87°/32.06°	37
*Q. variabilis*	ZJ_V	*Cerris*			191
*Q. fabri*	ZJ_F	*Quercus*			73
Total					301
*Q. acutissim*	YH_A	*Cerris*	Youhua village	118.02°/30.68°	50
*Q. chenii*	YH_C	*Cerris*			82
*Q. fabri*	YH_F	*Quercus*			41
Total					173

### 2.2 Leaf Phenotypic Trait Analysis

**FIGURE 1:**
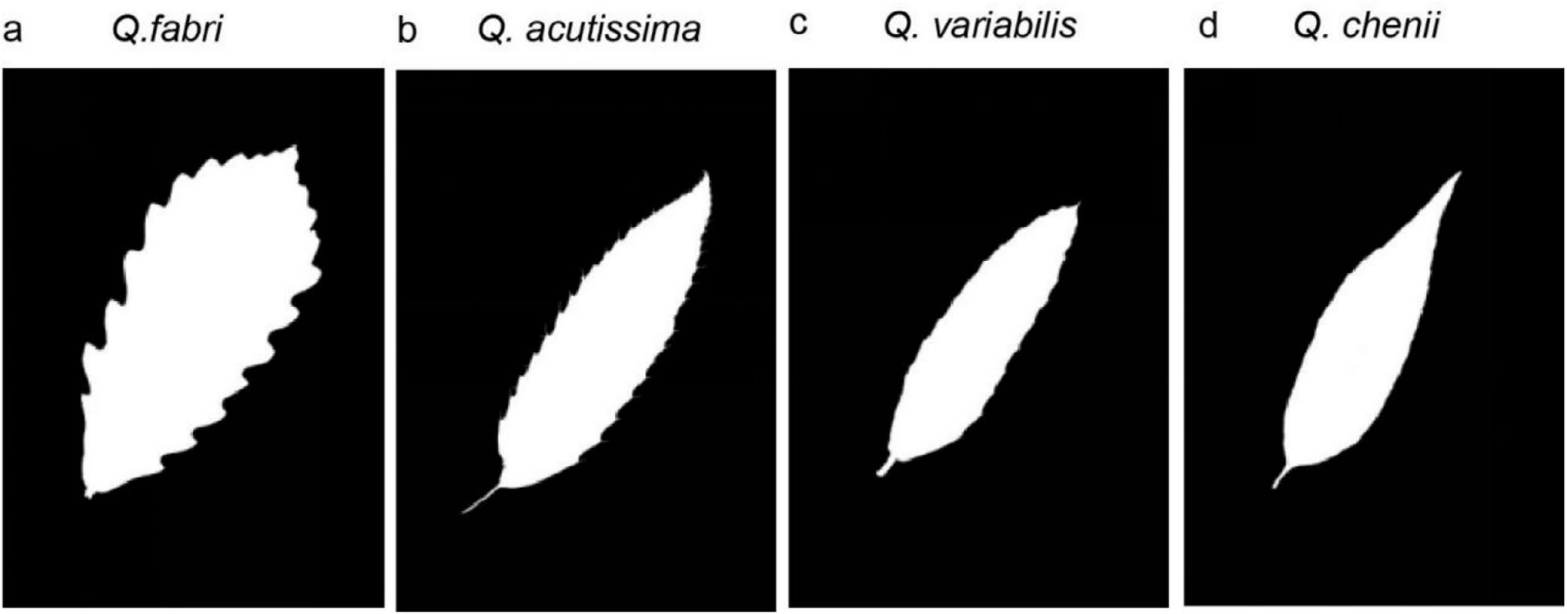
Image of leaves processed in Photoshop (a) *Quercus fabri*; (b) *Q. acutissima* ; (c) *Q. variabilis* ; (d) *Q. chenii* .

**FIGURE 2:**
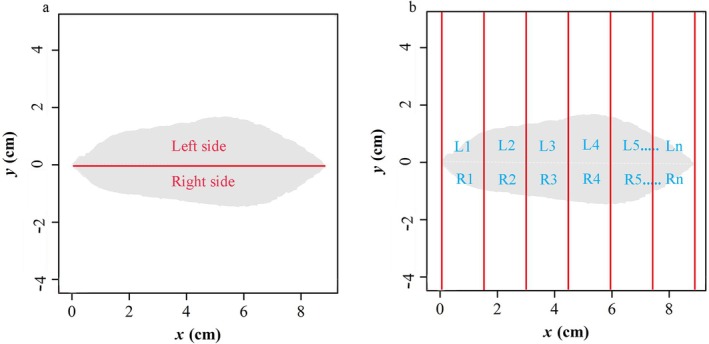
Illustration of the measure of bilateral symmetry for a blade of *Quercus* species: (a) Illustration of the areal ratio (AR) of the left‐ to the right‐side calculations; (b) The standardized index (SI) for measuring leaf fluctuating asymmetry.

The collected leaves were scanned using an EPSON V39 scanner, and the images were processed with Adobe Photoshop CC 2019 (Figure 1). Using the M‐file package in Matlab and the R script (BilatMeasure) developed by Shi et al. (2018), we extracted eight leaf morphological indices: leaf length (L_L_), width (W_L_), perimeter (P_L_), area (A_L_), left leaf area (A_Ll_), right leaf area (A_Lr_), leaf area ratio (AR), and standardized asymmetry index (SI) (Figure 2) (Shi et al. 2018; Li et al. 2024b).

### 2.3 Data Processing and Statistical Analysis

Leaf shape was quantified using width/length (RWL) and perimeter/area (RPA) ratios as follows:

(1) $$\mathrm{RWL}=W_{L}/L_{L}$$

(2) $$\mathrm{RPA}=P_{L}/A_{L}$$

FA serves as an indicator of developmental stability, measuring the variance between the leaves left and right sides. To assess fluctuating asymmetry (FA), we calculated the areal ratio (AR) and the standardized index (SI) (Figure 2) as follows:

(3) $$\mathrm{AR}=\frac{\Sigma_{\mathrm{i}=1}^{n}L_{i}}{\Sigma_{\mathrm{i}=1}^{n}R_{i}}$$

where *L*_*i*_ and *R*_*i*_ are the intersected left and right areas between the *i*‐th subregion, and

(4) $$\mathrm{SI}=\frac{1}{n}\sum_{i=1}^{n}\left|\frac{L_{i}-R_{i}}{L_{i}+R_{i}}\right|$$

where *n* equals 1000 in the calculation, is the average of the relative area differences among the *n* subregions.

When AR is converted into its natural logarithmic form, the degree of deviation of this logarithmic value from zero serves as a quantitative measure of FA. The greater this deviation, the higher the degree of FA. Similarly, when SI is transformed into its natural logarithmic equivalent, the magnitude of this logarithmic value directly correlates with the extent of FA. A larger logarithmic SI value indicates a higher level of FA, providing a precise and quantifiable metric for asymmetrical characteristics in leaf morphology.

The plasticity index (*PPI*) was calculated to assess environmental adaptability, using the formula:

$$\mathrm{PPI}=\left(P_{\max}-P_{\min}\right)/P_{\max}$$

where *P*_max_ and *P*_min_ represent the maximum and minimum values. A *PPI* close to 1 indicates high plasticity and adaptability to environmental changes. All statistical analyses were carried out using R software (version 4.1.0).

### 2.4 Statistical Analysis

A series of statistical methods were employed to investigate leaf trait variation across six populations from two regions and ten leaf morphological traits. Initially, the data were tested for normality and variance homogeneity, revealing deviations from normality and unequal variances. Given these findings, a Kruskal–Wallis test was conducted to assess significant differences in leaf traits between species and across regions, with Bonferroni correction applied to adjust for multiple comparisons, ensuring robust results. Subsequently, a mixed‐effects model was used to account for both fixed effects (species and region) and random effects (variability among populations within each region), providing a comprehensive understanding of the factors driving leaf trait variation. To assess the robustness of the results, Bootstrap resampling was employed to estimate confidence intervals and quantify uncertainty, offering a nonparametric approach to handle data variability. Finally, Principal Component Analysis (PCA) was performed to reduce dataset dimensionality, identify key patterns of variation in leaf traits, and visualize relationships between species, regions, and morphological characteristics. By sequentially applying these methods, we conducted a thorough examination of leaf trait variability across populations and regions, elucidating the ecological and biological factors influencing trait differentiation. All statistical analyses were carried out using R software (version 4.4.2).

## 3 Result

### 3.1 Leaf Phenotypic Traits Variation

In both Zijin Mountain and Youhua Village, *Q. acutissima* exhibited larger and more stable leaf area and leaf perimeter, while *Q. fabri* showed smaller areas and shorter perimeters with higher variability (Table 2). Specifically, *Q. acutissima* (ZJ_A and YH_A) had larger and more consistent leaf area and leaf perimeter compared to *Q. fabri* (ZJ_F and YH_F), which demonstrated greater variability in these traits. Similarly, for leaf length (L_L_) and leaf width (W_L_), *Q. acutissima* (ZJ_A and YH_A) showed larger and more consistent dimensions, while *Q. fabri* (ZJ_F and YH_F) exhibited shorter and more variable lengths and widths. *Q. acutissima* also had wider leaves in both regions, whereas *Q. fabri* showed narrower and more variable widths. In terms of width/length ratio (RWL) and perimeter/area ratio (RPA), *Q. acutissima* (ZJ_A and YH_A) had lower values, indicating broader leaves with smoother edges, while *Q. fabri* (ZJ_F and YH_F) had higher ratios, suggesting more complex leaf shapes. This trend was consistent across both regions, with *Q. fabri* exhibiting the highest variability in all traits, highlighting its more flexible adaptive strategy, particularly in Youhua Village. In summary, *Q. acutissima* consistently displayed larger, more stable leaf traits, while *Q. fabri* exhibited greater variability, reflecting differing adaptive strategies in response to their environments.

**TABLE 2:** Leaf phenotypic traits variation of six populations in two oak mixed forests.

Species	ZJ_A		ZJ_V		ZJ_F		YH_A		YH_C		YH_F		All species	
	Range	CV%	Range	CV%	Range	CV%	Range	CV%	Range	CV%	Range	CV%	Range	CV%
A_Ll_ (cm^2^)	12.37–88.15	34.40	1.47–75.00	29.41	1.48–59.31	67.18	7.44–62.82	29.29	0.11–63.36	32.55	2.52–162.09	114.71	0.11–162.09	48.09
A_Lr_ (cm^2^)	12.00–89.13	26.86	8.39–68.01	26.88	1.80–63.95	68.61	8.24–78.81	30.41	1.20–69.16	29.55	1.72–184.38	120.88	1.72–184.38	47.18
L_L_ (cm)	17.22–25.82	8.17	14.81–23.92	7.16	4.06–19.26	29.10	14.97–22.09	7.40	8.28–22.40	8.73	4.88–33.69	46.64	4.06–33.69	25.47
W_L_ (cm)	3.90–10.26	18.50	3.52–8.63	15.31	1.29–10.83	35.68	3.77–9.37	14.69	0.96–8.07	15.23	1.93–18.12	47.66	1.29–18.12	24.28
A_L_ (cm^2^)	50.54–177.28	22.30	41.28–121.18	17.32	4.55–120.13	65.55	43.52–114.39	17.07	1.52–95.35	17.04	7.71–328.21	114.45	4.55–328.21	41.12
P_L_ (cm)	46.71–73.59	9.32	42.61–68.56	6.46	12.21–62.39	29.73	43.62–76.72	9.44	25.86–73.36	9.73	16.14–114.79	45.47	12.21–114.79	23.21
RWL	0.19–0.49	20.31	0.19–0.55	18.37	0.24–0.69	14.34	0.23–0.54	16.13	0.09–0.94	21.11	0.25–0.7	17.54	0.15–0.94	26.23
RPA	0.40–1.08	19.05	0.45–1.19	16.72	0.49–3.26	37.20	0.49–1.11	16.32	0.55–1.77	24.2	0.29–2.21	33.94	0.29–3.26	37.36

Abbreviations: A_L_, area; A_Ll_, left leaf area; A_Lr_, right leaf area; AR, leaf area ratio; L_L_, leaf length; P_L_, perimeter; RPA, perimeter/area ratios; RWL, width/length ratios; W_L_, width.

Significant differences in leaf phenotypic traits were observed among the six populations in Zijin Mountain and Youhua Village, both among species and between regions (*p* < 0.001) (Table 3). These differences were confirmed not only through the Kruskal*–*Wallis test but also with the mixed‐effects model, which accounted for random effects such as population‐level variability (Tables S1–S10). It is worth noting that, according to the mixed‐effects model, *Q. fabri* exhibited significant differences in all eight leaf phenotypic traits when compared to the three species in the *Cerris* group. However, the three species within the *Cerris* group showed no significant differences in certain traits. Specifically, *Q. variabilis* showed no significant difference in A_Ll_ compared to other populations (Table S1), while *Q. acutissima* and *Q. chenii* had no significant differences in A_Lr_ (Table S2). Furthermore, both *Q. variabilis* and *Q. acutissima* did not show significant differences in W_L_ when compared to other populations (Table S4). Similarly, *Q. variabilis* did not show significant differences in A_L_ and P_L_ compared to other populations (Tables S5 and S6). Lastly, no significant differences were found in the RWL for *Q. acutissima* and in the RPA for *Q. fabri*, *Q. acutissima* , and *Q. variabilis* when compared to the other species (Table S7). Through the Bootstrap resampling results, significant variability was observed in the leaf phenotypic traits of *Q. fabri* compared to *Q. acutissima* , *Q. variabilis* , and *Q. chenii* , reflecting the species' greater adaptability to different environmental conditions (Table 4). Specifically, *Q. fabri* showed the widest confidence intervals for A_Ll_, with ranges from 2.79 to 39.43 cm^2^ in Zijin Mountain and 3.95 to 138.84 cm^2^ in Youhua Village, reflecting a high degree of variability in leaf size. *Q. fabri* also displayed greater variability in P_L_, with confidence intervals ranging from 15.47 to 50.49 cm in Zijin Mountain and 17.14 to 92.73 cm in Youhua Village, suggesting more flexibility in leaf perimeter compared to the relatively consistent perimeters of *Q. acutissima* and *Q. chenii* . Furthermore, for RWL, *Q. fabri* in Youhua Village showed higher values ranging from 0.31 to 0.68, indicating more complex leaf shapes and greater morphological variability compared to the lower RWL values of *Q. acutissima* , *Q. variabilis* , and *Q. chenii*. However, some leaf traits of *Q. fabri* exhibited smaller variability. For instance, in terms of L_L_, *Q. fabri* showed more stable leaf lengths, with confidence intervals from 5.10 to 16.62 cm, compared to the broader variability in leaf area and leaf perimeter. Similarly, *Q. fabri* showed relatively narrower variability in the RPA compared to *Q. chenii* , which exhibited more stable ratios.

**TABLE 3:** Comparative analysis of leaf phenotypic traits in two oak mixed forests.

	ZJ_A	ZJ_V	ZJ_F	YH_A	YH_C	YH_F	Total	*H*	Adjusted—*p*
	Mean ± SD	Mean ± SD	Mean ± SD	Mean ± SD	Mean ± SD	Mean ± SD	Mean ± SD		
A_Ll_ (cm^2^)	42.60 ± 14.65	36.68 ± 10.79	14.75 ± 9.91	35.72 ± 10.46	32.73 ± 10.51	22.70 ± 26.04	31.88 ± 15.35	780.78	< 0.000
A_Lr_ (cm^2^)	41.85 ± 11.24	37.45 ± 10.06	14.82 ± 10.17	35.83 ± 10.90	34.57 ± 10.06	23.84 ± 28.81	32.59 ± 15.4	812.74	< 0.001
L_L_ (cm)	20.91 ± 1.71	19.29 ± 1.38	9.92 ± 2.89	18.02 ± 1.33	17.66 ± 1.50	11.47 ± 5.35	16.95 ± 4.32	1336.90	< 0.001
W_L_ (cm)	6.23 ± 1.15	5.77 ± 0.88	4.51 ± 1.61	6.21 ± 0.91	6.07 ± 0.89	5.67 ± 2.70	5.7 ± 1.39	382.48	< 0.001
A_L_ (cm^2^)	84.44 ± 18.83	74.13 ± 12.84	29.57 ± 19.39	71.56 ± 12.22	67.30 ± 10.95	46.54 ± 53.26	64.48 ± 26.55	943.11	< 0.001
P_L_ (cm)	57.24 ± 5.34	52.22 ± 3.37	30.24 ± 8.99	51.37 ± 4.85	49.43 ± 4.67	35.64 ± 16.20	47.35 ± 11.0	1171.71	< 0.001
RWL	0.30 ± 0.06	0.30 ± 0.06	0.45 ± 0.06	0.35 ± 0.06	0.35 ± 0.07	0.50 ± 0.09	0.35 ± 0.09	1069.32	< 0.001
RPA	0.70 ± 0.13	0.72 ± 0.12	1.27 ± 0.47	0.73 ± 0.12	0.76 ± 0.18	1.05 ± 0.36	0.84 ± 0.47	665.91	< 0.001

*Note:* Phenotypic differences among the six populations were assessed using the *Kruskal–Wallis* test, with Bonferroni correction applied for multiple comparisons.

**TABLE 4:** Using bootstrap resampling to analyze differences in leaf morphology of 8 indices among six oak populations.

	ZJ_A	ZJ_V	ZJ_F	YH_A	YH_C	YH_F	Total
	CI (2.5%–97.5%)	CI (2.5%–97.5%)	CI (2.5%–97.5%)	CI (2.5%–97.5%)	CI (2.5%–97.5%)	CI (2.5%–97.5%)	CI (2.5%–97.5%)
A_Ll_ (cm^2^)	17.61–75.13	14.55–58.44	2.79–39.43	17.90–57.35	9.90–48.95	3.95–138.84	31.24–32.50
A_Lr_ (cm^2^)	20.25–63.08	16.78–58.14	3.23–40.10	15.01–55.61	13.46–54.86	4.60–95.47	31.95–33.24
L_L_ (cm)	17.53–24.58	16.51–22.00	5.10–16.62	15.42–20.59	14.85–20.78	5.83–29.32	16.76–17.12
W_L_ (cm)	4.03–8.49	4.12–7.67	1.93–8.56	4.58–8.33	4.11–7.57	2.48–14.42	5.64–5.76
A_L_ (cm^2^)	53.46–123.85	51.37–101.73	5.84–82.96	48.68–95.71	39.74–84.71	9.59–231.44	63.46–65.61
P_L_ (cm)	49.26–69.45	46.86–58.95	15.47–50.49	45.17–61.75	43.94–61.62	17.14–92.73	46.86–47.83
RWL	0.21–0.45	0.22–0.42	0.35–0.59	0.26–0.46	0.20–0.47	0.31–0.68	0.35–0.36
RPA	0.47–0.97	0.51–0.99	0.59–2.60	0.55–0.99	0.58–1.22	0.35–1.91	0.83–0.87

Abbreviations: A_L_, area; A_Ll_, left leaf area; A_Lr_, right leaf area; AR, leaf area ratio; CI, confidence interval; L_L_, leaf length; P_L_, perimeter; RPA, perimeter/area ratios; RWL, width/length ratios; W_L_, width.

### 3.2 Leaf Fluctuating Asymmetry and Plasticity Analysis

**FIGURE 3:**
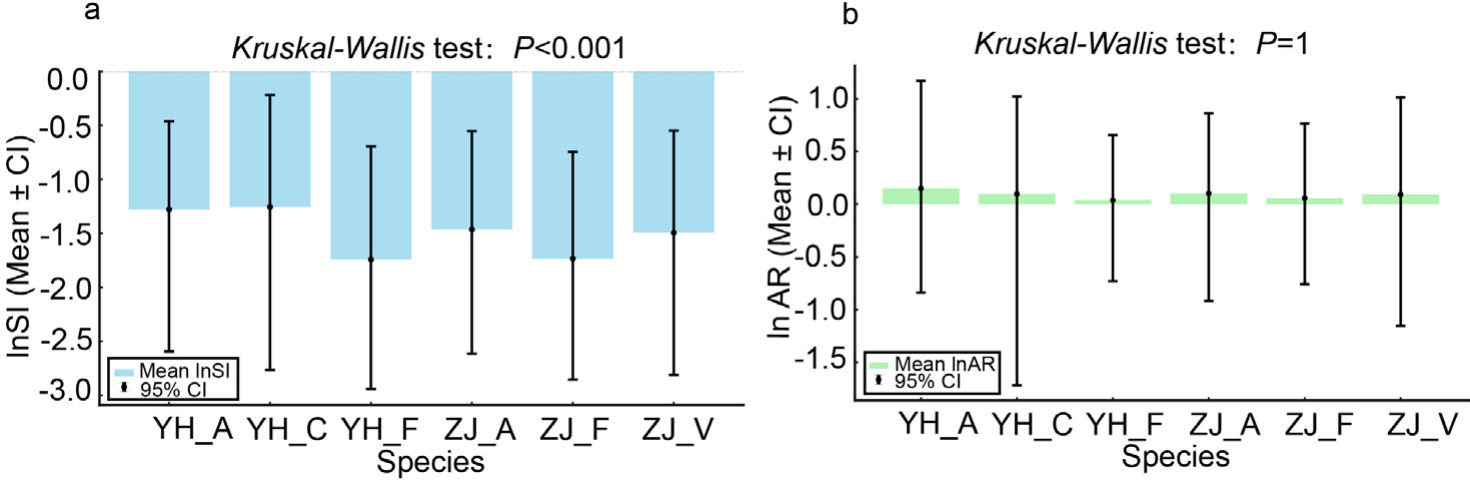
Leaf fluctuating asymmetry (FA) of six oak populations in two oak mixed forests: (a) The areal ratio (AR); (b) the standardized index (SI).

Figure 3 presents the analysis of leaf fluctuating asymmetry (FA) across six oak populations in Zijin Mountain and Youhua Village, focusing on the Standardized Index (SI) and the Areal Ratio (AR) in their natural logarithmic forms. The ln(SI) values of the six species are arranged in descending order as follows: YH_C > YH_A > ZJ_A > ZJ_V > ZJ_F > YH_F. A higher logarithmic SI value represents a higher level of FA. Therefore, the species are ranked by the degree of asymmetry in fluctuation from high to low as follows: YH_C > YH_A > ZJ_A > ZJ_V > ZJ_F > YH_F. We found that the differences in fluctuation asymmetry between species were highly significant through the *Kruskal–Wallis* test (*p* < 0.001). Additionally, the mixed‐effects model confirmed significant differences in FA among the six species (Tables S9 and S10), but the confidence intervals indicate that the range of FA variation across the six species is similar. A similar pattern can be observed in Figure 3b, where species from the *Cerris* group consistently have higher FA than the species from the *Quercus* group. The FA values are ranked from high to low as follows: YH_A > ZJ_A > YH_C > ZJ_V > ZJ_F > YH_F. However, the *Kruskal–Wallis* test found that the FA between species was not significant (*p* = 1), and the confidence intervals showed that the leaf variation range for the four species in the *Cerris* group was relatively large. Furthermore, we found that the FA in leaf shape for Youhua Village's oaks was higher than that of Zijin Mountain, especially for the *Cerris* species.

**FIGURE 4:**
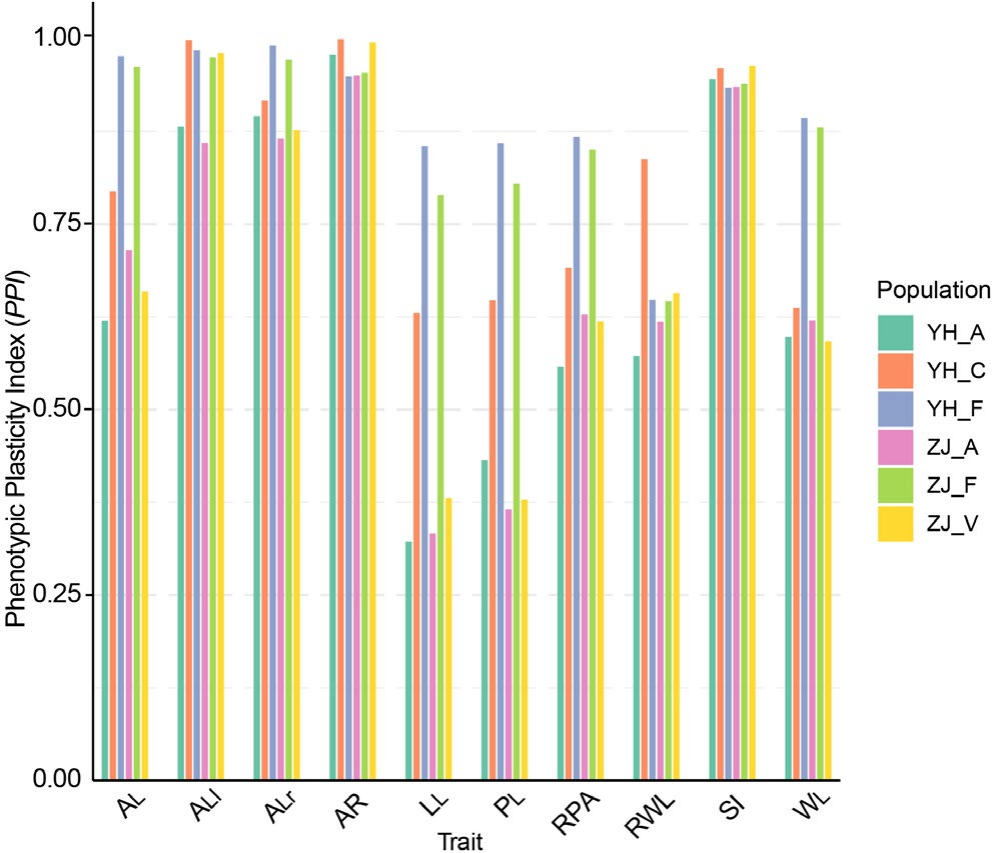
Comparative analysis of the plasticity of leaf phenotypic traits in two oak mixed forests. A_L_, area; AR, leaf area ratio; L_L_, Leaf length; A_Ll_, left leaf area; P_L_, perimeter; RPA, perimeter/area ratios; A_Lr_, right leaf area; AR, the areal ratio; SI, the standardized index; W_L_, width; RWL, width/length ratios.

Figure 4 presents a comparative analysis of the plasticity of leaf phenotypic traits in the six oak populations across Zijin Mountain and Youhua Village. The plasticity index (*PPI*) was used to measure the degree of variability in these traits under different environmental conditions. In the plasticity analysis, the trend observed in the *PPI* differed from that of FA. The two species of the *Quercus* group (YH_F, ZJ_F) exhibited higher plasticity in 6 out of the 10 leaf shape indicators (A_l_, L_L_, P_L_, RPA, W_L_, A_Lr_). Among these, *Quercus* species appeared to show greater leaf phenotypic plasticity. In contrast, species from the *Cerris* group ( *Q. variabilis* , *Q. chenii* ) showed higher significance in the remaining four leaf shape indicators (RWL, ALl, AR, and SI). When comparing the leaf phenotypic plasticity between the two regions, it is clear that species from Youhua Village exhibited higher overall leaf plasticity. This pattern aligns closely with the observed trend in FA.

### 3.3 Clustering Patterns of Oak Populations Based on Leaf Phenotypic Traits

**FIGURE 5:**
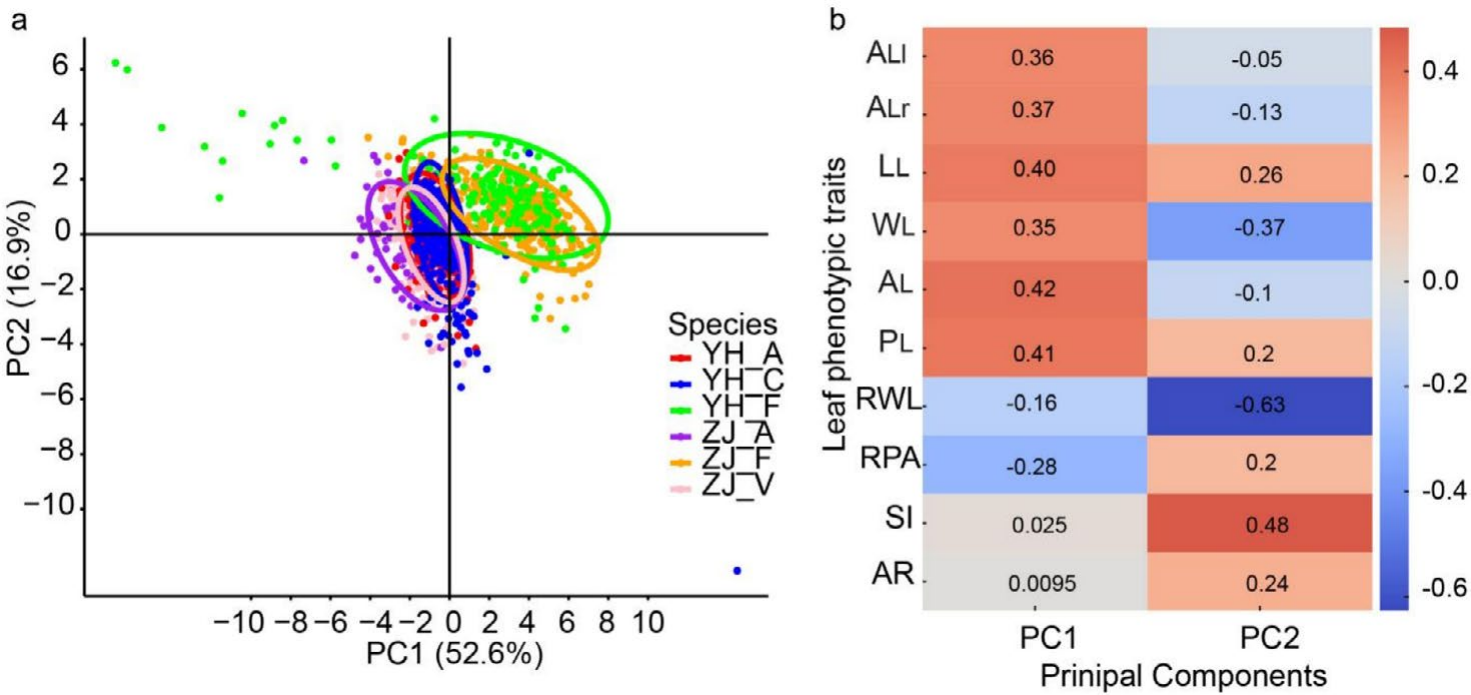
Clustering analysis of oak populations based on 10 leaf phenotypic traits (see M&M for traits code): (a) Two‐dimensional principal component analysis; (b) Correlation analysis. A_L_, area; A_Ll_, left leaf area; A_Lr_, right leaf area; AR, leaf area ratio; AR, the areal ratio; L_L_, Leaf length; P_L_, perimeter; RPA, perimeter/area ratios; RWL, width/length ratios; SI, the standardized index; W_L_, width.

According to Figure 5, the overall population clustering (left diagram) reveals that populations from the same region, such as *Q. acutissima* (ZJ_A) and *Q. variabilis* (ZJ_V) from Zijin Mountain, and *Q. chenii* (YH_C) and *Q. fabri* (YH_F) from Youhua Village, tend to cluster together. This indicates regional similarities in leaf traits. Additionally, species‐specific groupings are evident, with *Q. acutissima* from both regions clustering closely, suggesting consistent leaf traits across different environments. This pattern is also observed for *Q. variabilis* and *Q. fabri*, highlighting species‐specific trait consistency. The distinct clustering of populations from Zijin Mountain and Youhua Village underscores the significant influence of local environmental factors on leaf trait variability. Secondly, the trait clustering (right diagram) shows how specific leaf phenotypic traits group together across populations. Certain traits, like L_L_ and W_L_, cluster closely, reflecting similar patterns of variability and adaptation. In contrast, traits like AR and SI show distinct clustering, indicating unique patterns of fluctuating asymmetry. This clustering provides insights into the coordinated responses and specialized adaptations of the oak populations to environmental pressures. In summary, Figure 5 reveals regional and species‐specific clustering patterns based on leaf phenotypic traits, emphasizing the influence of local environmental conditions and genetic factors. The clustering of specific traits further elucidates the adaptive strategies and phenotypic plasticity of these oak populations.

## 4 Discussion

### 4.1 Species Variation in Leaf Traits and Adaptive Strategies

Our study reveals that species in the Cerris group, such as *Q. acutissima* , *Q. variabilis* , and *Q. chenii* , exhibit leaf phenotypic stability accompanied by higher fluctuating asymmetry (FA). FA is increasingly recognized as a sensitive indicator of developmental instability under environmental stress (Klingenberg 2019). In contrast, the species in the *Quercus* group, *Q. fabri*, demonstrate phenotypic instability with lower FA but higher plasticity. Phenotypic plasticity has been shown to play a critical role in plant adaptation to heterogeneous environments, particularly under climate change scenarios (Matesanz and Ramírez‐Valiente 2019). This finding reveals distinct adaptive strategies employed by different oak groups in response to environmental conditions (Matesanz and Ramírez‐Valiente 2019). Species in the *Cerris* group maintain leaf shape stability through genetic conservatism but are more sensitive to subtle environmental disturbances, resulting in higher FA (Mendes et al. 2018). This strategy likely corresponds to their adaptation in relatively stable environments, such as the artificial forests in Zijin Mountain, where stable phenotypic traits are advantageous (Levis and Pfennig 2019). However, this stability may be less effective in the face of environmental fluctuations, as their developmental robustness is lower, making them more susceptible to environmental stress (Pérez‐Pedraza et al. 2021). In contrast, *Q. fabri* from the *Quercus* group adapts to more variable environments by exhibiting high plasticity and developmental robustness, enabling it to adjust its leaf traits while maintaining low FA. This strategy confers a stronger adaptive capacity in the natural forests of Youhua Village, a more dynamic environment, where it can flexibly respond to diverse and uncertain environmental conditions while keeping FA at lower levels (Vaca‐Sánchez et al. 2021; Li, Wei, et al. 2021).

Furthermore, we compared the FA and phenotypic plasticity of oaks between the artificial forest on Zijin Mountain and the natural forest in Youhua Village. Our study found that oaks in the natural forest of Youhua Village exhibited higher FA and greater phenotypic plasticity, while oaks in the Zijin Mountain artificial forest showed lower FA and plasticity. The patterns of leaf variation in plants differ significantly across environments, reflecting the different adaptive strategies they employ in response to varying ecological conditions (Liu et al. 2023; Kormann et al. 2024; Gao et al. 2023; Solé‐Medina et al. 2022). The environment in the Zijin Mountain artificial forest is relatively stable, and human management may reduce environmental fluctuations, thereby decreasing the need for higher FA and plasticity in the oaks (Brockerhoff et al. 2013). In contrast, the environment in the Youhua Village natural forest is more complex and variable, requiring oaks to possess greater plasticity (Gao, Ji, et al. 2023) and developmental robustness to cope with environmental pressures (Mendes et al. 2018). Phenotypic plasticity and developmental robustness are key adaptive traits in heterogeneous environments, enabling species to respond to unpredictable environmental pressures (Gratani 2014; Levis and Pfennig 2019). For example, research on species like *Abutilon theophrasti* has shown that environments with limited resources or higher density enhance plastic responses, as plants need to adapt more flexibly to stressors (Wang and Zhou 2022). This suggests that the higher FA and plasticity observed in Youhua Village's natural forest may result from its more variable and competitive environmental conditions, where oaks are under stronger selective pressure to exhibit greater phenotypic flexibility. These findings support our hypothesis that phenotypic plasticity and FA are closely tied to environmental variability, with species adopting different adaptive strategies in response to diverse ecological pressures.

Additionally, we observed that species from the *Cerris* group exhibited higher FA in both forest types, while *Q. fabri* from the *Quercus* group showed higher phenotypic plasticity in both forest types. This suggests that different oak groups have developed distinct adaptive strategies during their evolutionary process. This phenomenon can be explained through the relationship between genetic co‐adaptation and FA. FA, as an indicator of developmental stability, is associated with genetic coadaptation, which is influenced by hybridization levels (Zakharov et al. 2020; Zakharov and Trofimov 2022). In our prior research, *Q. acutissima* and *Q. variabilis* had lower hybridization rates, whereas *Q. fabri* had a higher degree of hybridization, forming more F_2_ individuals (Li, Zhang, et al. 2021). This genetic background likely accounts for the observed FA differences. Genetic variation leads to greater phenotypic variation (Żabicka et al. 2023). At the same time, hybridization introduces new phenotypes, which can result in varying levels of phenotypic plasticity and influence the adaptive strategies of different species (Pérez‐Pedraza et al. 2021; Galán et al. 2024; Satokangas et al. 2023). Species in the *Cerris* group may be more inclined to maintain phenotypic stability through the stability of genetic networks, but their developmental processes may be more sensitive to environmental disturbances, limiting their performance in fluctuating environments. In contrast, *Q. fabri* from the *Quercus* group adapts to variable environments through high plasticity and developmental robustness, demonstrating a stronger capacity for environmental adaptation. As environmental changes and pressures increase, *Q. fabri* can maintain its growth and survival advantage through plastic responses, while species from the *Cerris* group may face greater challenges in adaptation. This reflects how species balance phenotypic stability, developmental robustness, and plasticity during their evolutionary process, although they may utilize different mechanisms to cope with environmental variability.

From both an ecological and evolutionary biology perspective, this phenomenon holds significant implications. Ecologically, it reflects the functional differences of different oak groups within ecosystems. Species in the *Cerris* group may be better suited for stable environments, occupying important ecological niches in stable ecosystems, whereas *Q. fabri* from the *Quercus* group exhibits stronger competitiveness and resource utilization abilities in variable environments, making it more adaptable. From an evolutionary biology perspective, studying the relationship between phenotypic stability, FA, and plasticity provides insights into how species balance stability and flexibility during evolution. Moreover, in forestry practices, understanding the phenotypic plasticity and developmental robustness of oak species can guide the selection of varieties adapted to different environments (Mátyás 2021). For instance, in stable artificial forest environments, *Cerris* group species with stable phenotypes may be preferred, while in more variable natural forest environments, *Quercus* group species with high plasticity may be chosen to improve oak growth performance and ecological function under different environmental conditions. This dual perspective underscores the importance of considering both ecological roles and evolutionary strategies when managing and conserving oak species, ensuring their resilience and adaptability in the face of environmental changes.

### 4.2 Clustering Analysis and Species Trait Consistency

The clustering analysis in Figure 5 reveals key insights into the relationship between leaf traits and species identification. The overall population clustering (left diagram) demonstrates that populations from the same region, such as *Q. acutissima* (ZJ_A) and *Q. variabilis* (ZJ_V) from Zijin Mountain, and *Q. chenii* (YH_C) and *Q. fabri* (YH_F) from Youhua Village, tend to cluster together, indicating regional similarities in leaf traits. This pattern highlights the significant influence of local environmental factors on leaf trait variability, suggesting that species in the same region share similar ecological pressures that shape their morphology. This regional clustering underscores the role of environmental conditions in driving phenotypic convergence among species, reflecting their adaptive responses to shared ecological challenges. Such findings emphasize the importance of considering local environmental contexts when studying species traits and their ecological adaptations.

However, the clustering also reveals a potential challenge for species identification: despite being from different regions, *Q. acutissima* from both Zijin Mountain and Youhua Village cluster closely. This suggests that *Q. acutissima* maintains consistent leaf traits across different environments, making it easier to identify as a distinct species. In contrast, *Q. fabri*, which shows greater morphological variability, forms a more dispersed cluster, reflecting its phenotypic plasticity and ability to adapt to diverse environmental conditions. This plasticity, while beneficial for survival in fluctuating environments, complicates the task of accurately identifying the species, as its leaf traits overlap with those of other species. This contrast highlights the trade‐off between phenotypic stability and plasticity in species identification. While *Q. acutissima* 's consistent traits facilitate clear taxonomic classification, *Q. fabri*'s adaptive variability underscores the challenges of identifying species with high plasticity, particularly in environments where trait overlap with other species is more likely. These findings emphasize the need for integrating multiple traits and environmental contexts when identifying species with high phenotypic variability.

In summary, while the clustering analysis highlights the consistency of species‐specific traits, it also underscores the challenges in species identification when morphological traits exhibit significant variation, especially in species with high plasticity like *Q. fabri*. The regional clustering and overlapping traits suggest that, while clustering can help group populations based on environmental similarities, accurate species identification may require a broader set of characteristics or molecular data to account for trait variability. This emphasizes the importance of combining morphological, ecological, and genetic approaches to improve the accuracy of species identification, particularly for highly plastic species whose traits may overlap with those of other species in response to environmental pressures. Such an integrated approach ensures a more comprehensive understanding of species boundaries and their adaptive strategies.

## Author Contributions

**Xuan Li:** data curation (equal), formal analysis (equal), software (equal), writing – original draft (equal). **Xuxu Bao:** formal analysis (equal), software (equal). **Yongfu Li:** formal analysis (equal), software (equal). **Yousry A. El‐Kassaby:** writing – review and editing (equal). **Yanming Fang:** resources (equal), writing – review and editing (equal).

## Ethics Statement

The authors have nothing to report.

## Consent

The authors have nothing to report.

## Conflicts of Interest

The authors declare no conflicts of interest.

## Supporting information


Tables S1–S10

## Data Availability

The datasets are available from the DRYAD (https://doi.org/10.5061/dryad.8cz8w9h24).
