# Supplementary material for: Phenotypic Variation of Oak Species (Quercus spp.) Reveals Adaptive Strategies Across Natural and Semi‐Artificial Oak Stands
Source: Ecol Evol. 2025 Jun 16;15(6):e71217. doi: 10.1002/ece3.71217 (PMC12168227; doi:10.1002/ece3.71217)
Supplement: Supplementary file 1 — Tables S1–S10 [file ECE3-15-e71217-s001.docx]

**Supplementary information**

**Table S1**  Using mixed effects model to predict differences in leaf morphology of A_Ll_ index among six oak populations

|  | Coefficients | Std. Error | *t*-value | *P*r(>\|*t*\|) |
| --- | --- | --- | --- | --- |
| (Intercept) | 35.7230 | 0.8559 | 41.739 | < 2e-16 *** |
| YH_C | -3.0823 | 1.0822 | -2.848 | 0.00444 ** |
| YH_F | -13.0221 | 1.2753 | -10.211 | < 2e-16 *** |
| ZJ_A | 6.8723 | 1.3515 | 5.085 | 3.98e-07 *** |
| ZJ_F | -20.9722 | 1.1147 | -18.814 | < 2e-16 *** |
| ZJ_V | 0.9570 | 0.9536 | 1.004 | 0.31573 |
| Regression Statistics | | | | |
| Residual SE | 12.89 |  |  |  |
| Multiple-*R*^2^ | 0.2959 |  |  |  |
| Adjusted-*R*^2^ | 0.2943 |  |  |  |
| *F*-statistic | 185.2 |  |  |  |
| *P-*value | < 2.2e-16 |  |  |  |

**Table S2** Using mixed effects model to predict differences in leaf morphology of A_Lr_  index among six oak populations

|  | Coefficients | Std. Error | *t*-value | *P*r(>\|*t*\|) |
| --- | --- | --- | --- | --- |
| (Intercept) | 35.8345 | 0.8570 | 41.813 | < 2e-16 *** |
| YH_C | -1.3496 | 1.0837 | -1.245 | 0.2131 |
| YH_F | -11.9980 | 1.2770 | -9.395 | < 2e-16 *** |
| ZJ_A | 6.0119 | 1.3533 | 4.443 | 9.33e-07 *** |
| ZJ_F | -21.0116 | 1.1162 | -18.824 | < 2e-16 *** |
| ZJ_V | 1.6107 | 0.9549 | 1.687 | 0.0918 |
| Regression Statistics | | | | |
| Residual SE | 12.91 |  |  |  |
| Multiple-*R*^2^ | 0.2982 |  |  |  |
| Adjusted-*R*^2^ | 0.2965 |  |  |  |
| *F*-statistic | 187.2 |  |  |  |
| *P-*value | < 2.2e-16 |  |  |  |

**Table S3** Using mixed effects model to predict differences in leaf morphology of L_L_ index among six oak populations

|  | Coefficients | Std. Error | *t*-value | *P*r(>\|*t*\|) |
| --- | --- | --- | --- | --- |
| (Intercept) | 18.0194 | 0.1519 | 118.656 | < 2e-16 *** |
| YH_C | -0.3808 | 0.1920 | -1.983 | 0.0475* |
| YH_F | -6.5448 | 0.2263 | -28.922 | < 2e-16 *** |
| ZJ_A | 2.8899 | 0.2398 | 12.051 | < 2e-16 *** |
| ZJ_F | -8.0978 | 0.1978 | -40.942 | < 2e-16 *** |
| ZJ_V | 1.2745 | 0.1692 | 7.532 | 7.25e-14 *** |
| Regression Statistics | | | | |
| Residual SE | 2.288 |  |  |  |
| Multiple-*R*^2^ | 0.72 |  |  |  |
| Adjusted-*R*^2^ | 0.7193 |  |  |  |
| *F*-statistic | 1133 |  |  |  |
| *P-*value | < 2.2e-16 |  |  |  |

**Table S4** Using mixed effects model to predict differences in leaf morphology of W_L_ index among six oak populations

|  | Coefficients | Std. Error | *t*-value | *P*r(>\|*t*\|) |
| --- | --- | --- | --- | --- |
| (Intercept) | 6.21368 | 0.08519 | 72.937 | < 2e-16 *** |
| YH_C | -0.15798 | 0.10773 | -1.467 | 0.143 |
| YH_F | -0.54785 | 0.12695 | -4.316 | 1.66e-05*** |
| ZJ_A | 0.02114 | 0.13452 | 0.157 | 0.875 |
| ZJ_F | -1.70836 | 0.11096 | -15.397 | < 2e-16 *** |
| ZJ_V | -0.44854 | 0.09492 | -4.725 | 2.44e-06 *** |
| Regression Statistics | | | | |
| Residual SE | 1.284 |  |  |  |
| Multiple-*R*^2^ | 0.146 |  |  |  |
| Adjusted-*R*^2^ | 0.1441 |  |  |  |
| *F*-statistic | 75.36 |  |  |  |
| *P-*value | < 2.2e-16 |  |  |  |

**Table S5** Using mixed effects model to predict differences in leaf morphology of A_L_ index among six oak populations

|  | Coefficients | Std. Error | *t*-value | *P*r(>\|*t*\|) |
| --- | --- | --- | --- | --- |
| (Intercept) | 71.557 | 1.370 | 52.249 | < 2e-16 *** |
| YH_C | -4.432 | 1.732 | -2.559 | 0.0106* |
| YH_F | -25.020 | 2.041 | -12.260 | < 2e-16 *** |
| ZJ_A | 12.884 | 2.163 | 5.958 | 2.97e-09*** |
| ZJ_F | -41.984 | 1.784 | -23.537 | < 2e-16 *** |
| ZJ_V | 2.568 | 1.526 | 1.683 | 0.0926 |
| Regression Statistics | | | | |
| Residual SE | 20.63 |  |  |  |
| Multiple-*R*^2^ | 0.3975 |  |  |  |
| Adjusted-*R*^2^ | 0.3961 |  |  |  |
| *F*-statistic | 290.8 |  |  |  |
| *P-*value | < 2.2e-16 |  |  |  |

**Table S6** Using mixed effects model to predict differences in leaf morphology of P_L_ index among six oak populations

|  | Coefficients | Std. Error | *t*-value | *P*r(>\|*t*\|) |
| --- | --- | --- | --- | --- |
| (Intercept) | 51.3728 | 0.4556 | 112.751 | < 2e-16 *** |
| YH_C | -1.9990 | 0.5761 | -3.470 | 0.000531*** |
| YH_F | -15.7368 | 0.6789 | -23.178 | < 2e-16 *** |
| ZJ_A | 5.8663 | 0.7195 | 8.154 | 5.86e-16*** |
| ZJ_F | -21.1311 | 0.5934 | -35.609 | < 2e-16 *** |
| ZJ_V | 0.8428 | 0.5077 | 1.660 | 0.097047 |
| Regression Statistics | | | | |
| Residual SE | 6.865 |  |  |  |
| Multiple-*R*^2^ | 0.6112 |  |  |  |
| Adjusted-*R*^2^ | 0.6103 |  |  |  |
| *F*-statistic | 693 |  |  |  |
| *P-*value | < 2.2e-16 |  |  |  |

**Table S7** Using mixed effects model to predict differences in leaf morphology of RWL index among six oak populations

|  | Coefficients | Std. Error | *t*-value | *P*r(>\|*t*\|) |
| --- | --- | --- | --- | --- |
| (Intercept) | 0.346451 | 0.004227 | 81.968 | < 2e-16 *** |
| YH_C | 0.001241 | 0.005345 | 0.232 | 0.816 |
| YH_F | 0.151568 | 0.006298 | 24.065 | < 2e-16 *** |
| ZJ_A | -0.046518 | 0.006674 | -6.970 | 4.17e-16*** |
| ZJ_F | 0.103617 | 0.005505 | 18.822 | < 2e-16 *** |
| ZJ_V | -0.045550 | 0.004709 | -9.672 | < 2e-16 *** |
| Regression Statistics | | | | |
| Residual SE | 0.06368 |  |  |  |
| Multiple-*R*^2^ | 0.5276 |  |  |  |
| Adjusted-*R*^2^ | 0.5265 |  |  |  |
| *F*-statistic | 492.3 |  |  |  |
| *P-*value | < 2.2e-16 |  |  |  |

**Table S8** Using mixed effects model to predict differences in leaf morphology of RPA index among six oak populations

|  | Coefficients | Std. Error | *t*-value | *P*r(>\|*t*\|) |
| --- | --- | --- | --- | --- |
| (Intercept) | 0.73470 | 0.02869 | 25.610 | < 2e-16 *** |
| YH_C | 0.06995 | 0.03628 | 1.928 | 0.0539 |
| YH_F | 0.31566 | 0.04275 | 7.384 | 2.16e-13*** |
| ZJ_A | -0.03105 | 0.04530 | -0.685 | 0.4932 |
| ZJ_F | 0.53124 | 0.03736 | 14.218 | < 2e-16 *** |
| ZJ_V | -0.01129 | 0.03196 | -0.353 | 0.7241 |
| Regression Statistics | | | | |
| Residual SE | 0.4322 |  |  |  |
| Multiple-*R*^2^ | 0.1726 |  |  |  |
| Adjusted-*R*^2^ | 0.1708 |  |  |  |
| *F*-statistic | 91.98 |  |  |  |
| *P-*value | < 2.2e-16 |  |  |  |

**Table S9** Using mixed effects model to predict differences in leaf morphology of SI index among six oak populations

|  | Coefficients | Std. Error | *t*-value | *P*r(>\|*t*\|) |
| --- | --- | --- | --- | --- |
| (Intercept) | 0.278472 | 0.009431 | 29.528 | < 2e-16 *** |
| YH_C | 0.006653 | 0.011925 | 0.558 | 0.57700 |
| YH_F | -0.103375 | 0.014053 | -7.356 | 2.66e-13*** |
| ZJ_A | -0.047108 | 0.014892 | -3.163 | 0.00158** |
| ZJ_F | -0.101851 | 0.012283 | -8.292 | < 2e-16 *** |
| ZJ_V | -0.054128 | 0.010508 | -5.151 | 2.82e-07*** |
| Regression Statistics | | | | |
| Residual SE | 0.1421 |  |  |  |
| Multiple-*R*^2^ | 0.06718 |  |  |  |
| Adjusted-*R*^2^ | 0.06506 |  |  |  |
| *F*-statistic | 31.75 |  |  |  |
| *P-*value | < 2.2e-16 |  |  |  |

**Table S10** Using mixed effects model to predict differences in leaf morphology of AR index among six oak populations

|  | Coefficients | Std. Error | *t*-value | *P*r(>\|*t*\|) |
| --- | --- | --- | --- | --- |
| (Intercept) | 1.16211 | 0.03870 | 30.030 | < 2e-16 *** |
| YH_C | -0.06097 | 0.04893 | -1.246 | 0.2129 |
| YH_F | -0.12384 | 0.05767 | -2.148 | 0.3667*** |
| ZJ_A | -0.05517 | 0.06111 | -0.903 | 0.3667 |
| ZJ_F | -0.10247 | 0.05040 | -2.033 | 0.0422* |
| ZJ_V | -0.06601 | 0.04312 | -1.531 | 0.1259 |
| Regression Statistics | | | | |
| Residual SE | 0.5831 |  |  |  |
| Multiple-*R*^2^ | 0.002748 |  |  |  |
| Adjusted-*R*^2^ | 0.0004854 |  |  |  |
| *F*-statistic | 1.215 |  |  |  |
| *P-*value | < 0.2996 |  |  |  |
